# Fast-growing immature ovarian teratoma during pregnancy: a case report and a review of the literature

**DOI:** 10.1186/s12884-022-04857-y

**Published:** 2022-06-27

**Authors:** Zuoxi He, Yukun Lu, Chuan Xie

**Affiliations:** 1 Department of Gynecology and Obstetrics, West China Second University Hospital, Sichuan University, Sichuan Province, No. 20 Section Three, South Renmin Road, Chengdu, 610041 People’s Republic of China; 2 Key Laboratory of Birth Defects and Related Diseases of Women and Children, Ministry of Education, Sichuan Province, Chengdu, People’s Republic of China

**Keywords:** Immature teratoma, Teratoma, Pregnancy, Surgery, Chemotherapy

## Abstract

**Background:**

Immature ovarian teratoma is one of the three common malignant ovarian germ cell tumors. However, immature ovarian teratoma in pregnancy is very rare. Due to the rare occurrence, there is little evidence regarding its diagnosis, optimal management, and prognosis. Hence, we present a case of immature teratoma diagnosed during pregnancy, and analyze its clinicopathological features, management and prognosis.

**Case presentation:**

A 28-year-old woman underwent a sonographic examination revealed no abnormality in the bilateral adnexal area before 29 weeks gestational age (WGA). At 29 WGA, ultrasound demonstrated a 9.7 × 8.5 × 6.4 cm complex structure in the left adnexal area. At 30 WGA, repeated ultrasound revealed rapid growth of tumor mass, measuring 25.0 × 15.0 × 13.7 cm. An elective cesarean section combined with exploratory laparotomy was performed at 33 WGA. Intraoperative frozen pathological examination suggested left ovarian immature teratoma. Then, she underwent a complete staging surgery. Subsequently, the patient received 4 cycles of bleomycin-etoposide-cisplatin (BEP) chemotherapy. After 18 months of follow-up, there is no sign of tumor recurrence till now.

**Conclusions:**

This case report suggests that the benefits and risks of timely treatment for patients and fetuses should be fully assessed by a multidisciplinary team. The early diagnosis, the timing of surgery and chemotherapy, the choice of chemotherapy for BEP will determine the prognosis. Surgery and combination chemotherapy with BEP play an important role in the treatment of immature teratomas in pregnancy, and could gain successful and satisfactory outcomes for mother and fetus.

## Background

The incidence of ovarian tumors in pregnancy is relatively low, and nearly about 0.2–3.8 cases in every 100,000 pregnancies are associated with ovarian cancer [1]. Malignant ovarian germ cell tumor (MOGCT) originates from ovarian primordial cell, accounting for 2–3% of ovarian malignant tumors [2]. The main histological types of MOGCT include asexual cell tumor, yolk sac tumor and immature teratoma. Immature teratoma is the third most common histological type of MOGCT. However, the incidence of immature ovarian teratoma during pregnancy is very rare. It has been reported that immature teratoma in pregnancy is estimated to account for approximately 1% of all teratomas [3].

Due to the rare occurrence, little evidence exists on the diagnosis, optimal management and prognosis of immature teratoma diagnosed in pregnancy. Hence, we present a case of immature teratoma diagnosed during pregnancy, and analyze its clinicopathological features, management and prognosis.

## Case presentation

**Fig. 1:**
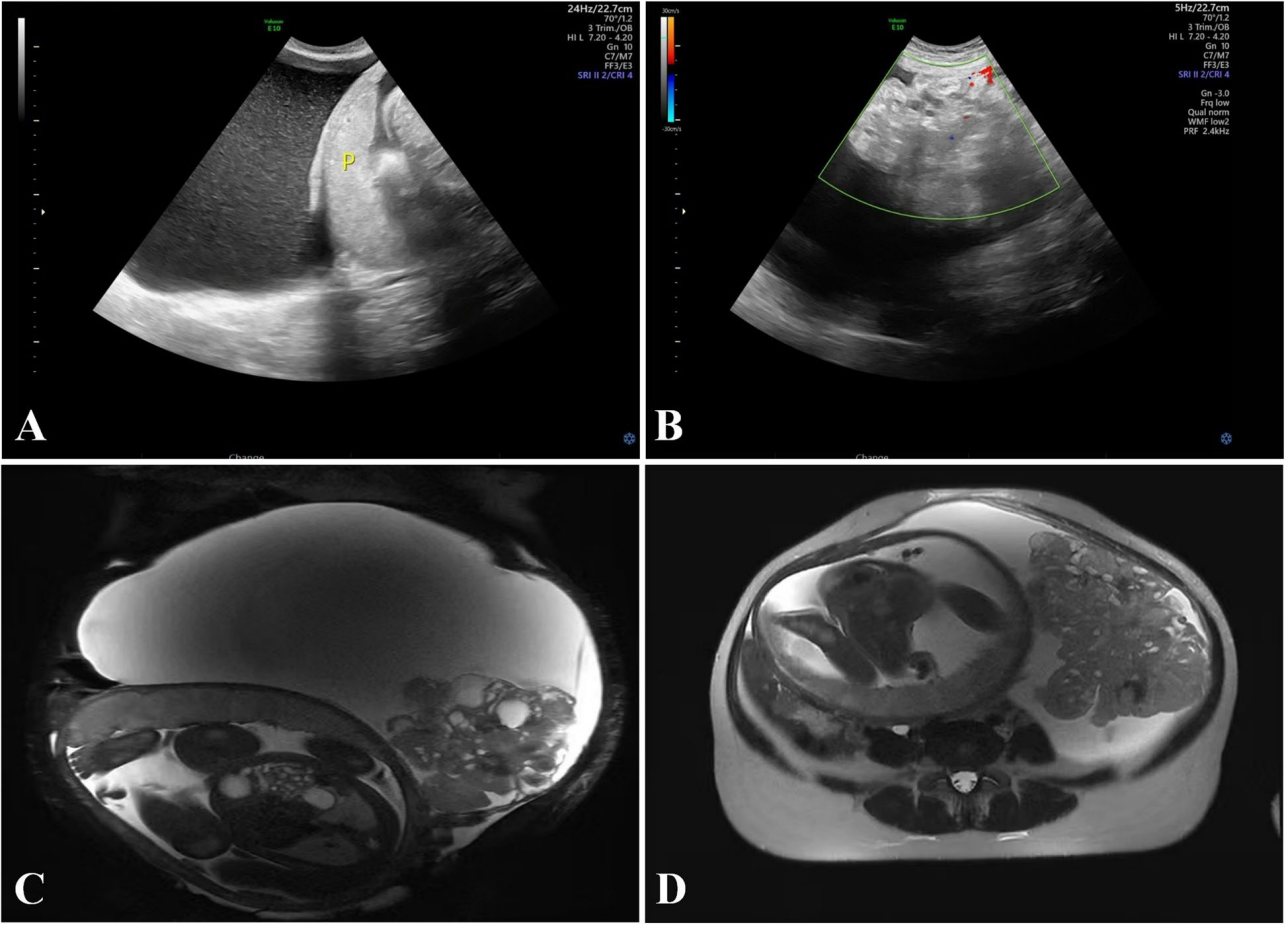
The ultrasound and MRI images of the tumor. **A** and **B**, sonographic examination demonstrated there was a 25.0 × 15.0 × 13.7 cm cyst in the left adnexal area, and the cyst was found to have a 15.7 × 11.3 × 14.1 cm heterogeneous echogenicity, which was irregular cauliflower-shaped and had strong blood flow. p indicate placenta. **C** and **D**, Abdominal magnetic resonance imaging (MRI) revealed a 30 × 17.4 × 18.9 cm cystic and solid mass on the left side of the pelvic cavity, and the solid component was irregular-shaped and cauliflower-shaped appearance

**Fig. 2:**
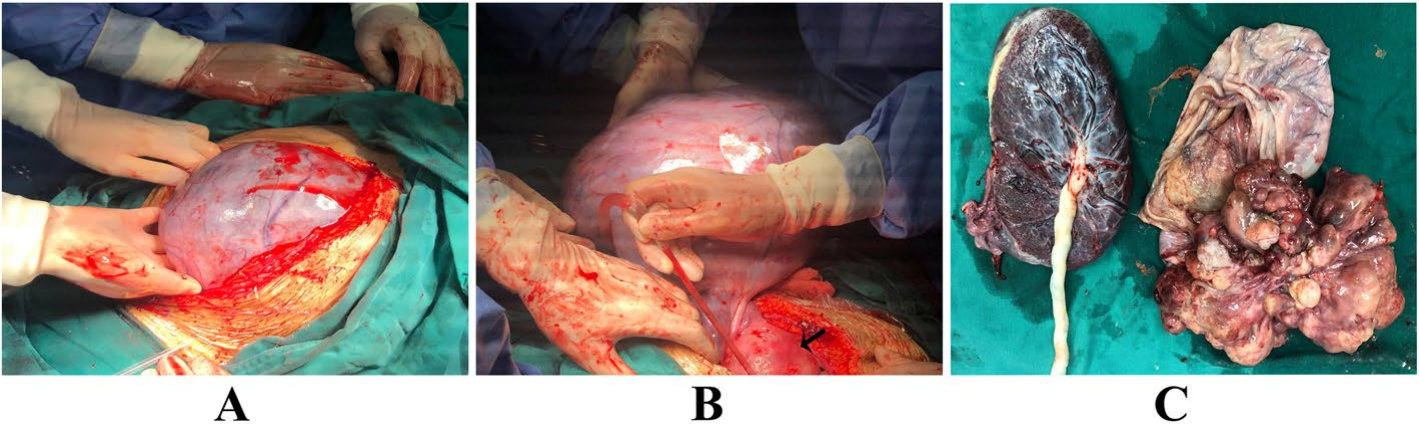
A giant immature teratoma of the left ovary. **A**, After entering the abdomen, there was a huge mass with a smooth surface in the pelvic and abdominal cavity. **B**, Intraoperative exploration revealed that the mass originated from the left ovary. The black arrow in **B** points to the uterus after cesarean section. **C**, Gross examination of the excised specimen showed the tumor mass was cystic and solid

A 28-year-old female patient, gravida 1 para 0 without special medical history underwent a series of routine sonographic examinations during the first and second trimesters of the pregnancy. All these examinations before 29 weeks’ gestational age revealed normal fetal growth and no obvious abnormality in the bilateral adnexal area. The patient did not show any typical symptoms and discomfort before 29 weeks’ gestational age, but She complained of occasional mild abdominal pain after the gestational age of 29 weeks. At 29 weeks’ gestational age, routine obstetric ultrasound demonstrated a 9.7 × 8.5 × 6.4 cm complex structure in the left adnexal area. Blood tumor markers including carbohydrate antigen-125 (CA-125) and alpha-fetoprotein (AFP) were in the normal range. At the gestational age of 30 weeks, repeated abdominal ultrasound revealed rapid growth of tumor mass, measuring 25.0 × 15.0 × 13.7 cm. The cyst was found to have a 15.7 × 11.3 × 14.1 cm heterogeneous echogenicity, which was irregular cauliflower-shaped and had strong blood flow. Sonographic examination showed no ascites or pleural effusion (Fig. 1 A and B). Abdominal magnetic resonance imaging (MRI) revealed a 30 × 17.4 × 18.9 cm cystic and solid mass on the left side of the pelvic cavity, and the solid component was irregular-shaped and cauliflower-shaped appearance (Fig. 1 C and D). Blood tumor markers were as follows: CA-125 of 43.4 U/mL and AFP of 173.4 ng/mL. We used the international ovarian tumor analysis (IOTA) simple rules to evaluate the mass, and the tumor was classified as malignant according to IOTA simple rules. Maternal–fetal medicine, gynecologic oncology and neonatal intensivist consults were obtained. Due to rapid growth of the masses, and concern for malignancy, an elective cesarean section combined with exploratory laparotomy was recommended after dexamethasone was given to the mother to accelerate fetal lung maturation. She underwent an elective cesarean section at 33 weeks’ gestational age. A female infant was delivered (Apgar Score 9–10-10, weight 1610 g). Intraoperative findings revealed there was a solid and cystic mass measuring 35 × 20 × 15 cm in diameter with smooth surface, arising from the left ovary (Fig. 2 A and B). The capsule of the tumor mass was intact, and there was no tumor infiltration in the left fallopian tube and the right adnexa. After careful examination of the pelvic and abdominal peritoneum and other organ surfaces (including the omentum, diaphragm, liver, and stomach), no suspicious invasive lesions were found. Gross examination revealed that the tumor was composed of cystic and solid components (Fig. 2 C), and the wall of cystic component was thin and smooth, containing yellowish clear liquid. The tumor mass contained cauliflower-like solid components, measuring 18 × 17 × 15 cm (Fig. 2 C). The intraoperative frozen pathological examination suggested left ovarian immature teratoma. Then, she underwent a left adnexectomy, pelvic mass resection, right ovarian biopsy, bilateral pelvic lymph node dissection, para-aortic lymph node sampling, and omentectomy. Peritoneal washings were also collected and sent for pathological examination. The final histological and immunohistochemical results confirmed the diagnosis of immature left ovarian teratoma (WHO II grade). The tumor was limited to the left ovary without involvement of the left fallopian tube, and no tumor infiltration was found in the omentum, pelvic and paraaortic lymph nodes. Tumor cells were also not observed in peritoneal washings. The tumor was staged as FIGO I C1 (the tumor was limited to the left ovary with surgical spill). After evaluation by oncologists, post-operative chemotherapy was recommended. Therefore, she underwent 4 cycles of BEP chemotherapy regimen. After 18 months of follow-up, there is no sign of tumor recurrence till now.

## Discussion

Immature teratomas are teratomas that contain components of embryonal neuroectodermal tissue. Immature ovarian teratomas account for approximately 1–3% of ovarian malignancy, and they frequently occur in young women [2]. The incidence of immature teratoma cases diagnosed during pregnancy is estimated to be about 1% of all ovarian teratomas in young and reproductive period [3]. The incidence of immature teratomas in pregnancy is very rare, so there is no clear consensus about its management strategy. Multidisciplinary collaboration including gynecologic oncologist, pathologist, maternal–fetal medicine is needed for early diagnosis, optimal management, management of delivery and choice of chemotherapy. This case report discusses the diagnosis, management strategy, and prognosis of immature teratoma in pregnancy.

Immature teratomas in pregnancy, like other immature teratomas, are almost asymptomatic when the tumor mass is relatively small, so it is difficult to early diagnose by symptom. The majority of immature teratomas in pregnancy are found by routine obstetric ultrasound. In this case report, immature teratomas were first detected by routine obstetric ultrasound. In addition to ultrasound, serum tumor markers are useful in the diagnosis of immature teratomas. Although, the elevation of serum tumor markers in pregnancy is usually associated with the physiology of pregnancy itself, we often find the increasing of alpha-fetoprotein (AFP), carcinoembryonic antigen (CEA), lactate dehydrogenase (LDH), carbohydrate antigen-125 (CA-125) and carbohydrate antigen 19–9 (CA19-9) in pregnant patients with immature teratoma. Patient in this case report was detected to have elevated serum AFP and CA-125. As can be seen from Table 1, the elevation of AFP, CA 19–9, LDH and/or CA-125 can be found in eight patients for whom the data of serum tumor markers was available. It was reported that ultrasound for diagnosis of immature ovarian teratomas has specificity 87%, sensitivity 90%, negative predictive value 97% and positive predictive value 69% in define of suspected ovarian malignancy [3, 4]. Magnetic Resonance Imaging (MRI) is suggested to evaluate the metastatic lesions in abdominal cavity when the ultrasound diagnosis is uncertain. MRI using contrast gadoliniumis proved to be safe for fetus in the second and third trimester of pregnancy [5]. Although there are many inspection methods to find immature teratoma in pregnancy, its definite diagnosis depends on postoperative pathological examination.

**Table1:** Reported immature ovarian teratoma in pregnancy

Case	Author	Age, y	GA at discovery	Tumor Size (at discovery)	CT regimen /GA at start /cycle(s)	Operation/GA	Stage	Delivery mode/GA (at delivery)	Tumor Size (at operation)	Tumor marker^a^	Fetal outcome	Maternal outcome
1	Hassan A A et al. 1984 [6]	28	28w	N/A	Vincristine + actinomycin D + cyclophosphamide/Postpartum/1	Right salpingo-ophorectomy + partial omentectomy/29w	N/A	CS/29w	22 × 20 × 11 cm	N/A	1050 g	Died 3 months after the operation
2	Christman J E et al. 1990 [7]	29	6w	18 × 20 cm	Cisplatin + vinblastine + bleomycin/19w/1 Cisplatin + vinblastine + bleomycin/Pos-tpartum/3	Right salpingo-oophorectomy + surgical staging procedure /15w Peritoneal washings + peritoneal biopsies + hysterectomy + omentectomy + pelvic and paraaortic lymphadenectomy/Po-stpartum	IC	VD/≧37w	N/A	N/A	Male,3232 g Apgar 8,9 (1st, 5th min), normal Follow-up good	52 months after CT follow-up good
3	Poremba C et al. 1993 [8]	27	38w	8 × 6 × 4.5 cm	N/A	N/A	N/A	CS/38w	8 × 6 × 4.5 cm	N/A	Female,hydroceph-alus(intracranial teratoma) The fetal survived only 9 weeks	N/A
4	Horbelt D et al. 1994 [9]	18	18w	5.2 × 9.4 × 8.6 cm	BEP/21w/3	Right oophorectomy + infracolic mentectomy + perit-oneal biopsies /20w	IA	VD/39w	236 g	AFP 477.8 IU / mL (18w)	Female,2769 g Apgar 4,7(1st, 5th min) Anemia after delivery Normal development	N/A
5	O'Connor D M,et al. 1994 [10]	N/A	N/A	N/A	N/A	N/A	I	N/A	N/A	N/A	N/A	N/A
6		N/A	N/A	N/A	N/A	N/A	I	N/A	N/A	N/A	N/A	N/A
7		N/A	N/A	N/A	N/A	N/A	I	N/A	N/A	N/A	N/A	N/A
8	Whitecar M P et al. 1999 [11]	N/A	N/A	N/A	N/A	N/A	N/A	N/A	N/A	N/A	N/A	N/A
9	Bakri Y N 2000 [12]	21	8w	N/A	N/A	Total abdominal hysterectomy + bilateral salpingo-oophorectomy/N/A	III Undetermined?	N/A	N/A	N/A	N/A	Died at the second trimester
10	Kishimoto K et al. 2002 [13]	28	35w	18 × 17 × 11 cm	Receive five cycles of CT postpartum (the regimen is not recorded)	Abdominal total simple hysterectomy + bilateral salpingo- ophrectomy + pelvic and paraaortic lymphadenectomy + partial omentectomy/N/A	IIIC	CS/≧38w	20 × 15 × 13 cm	AFP 830.1 ng/ml(35w)	2308 g and the post-operative course was uneventful	Remains alive 9 months after delivery
11	Agarwal N et al. 2003 [14]	N/A	N/A	N/A	N/A	Excision of growth /33w	NA	CS/33w	N/A	N/A	N/A	N/A
12	Han J Y et al. 2005 [15]	27	24w	6 × 5 cm	BEP/30w/2 BEP/Postpartum/3	Right salpingo-oophorectomy /26w Laparoscopic dissection of bilateral pelvic + paraaortic lymph nodes + omentectomy + biopsy of left ovary/Postpartum	IA	VD/38w	N/A	AFP 268 IU/ml(16w)	Male, 2970 g Apgar score 9–10(1st, 5th min) 26 months after follow-up normal physiological and neurological development	26 months of follow-up and no evidence of malignant tumor
13	Zhao X Y et al. 2006 [16]	24	17 + w	N/A	N/A	Left salpingo-oophorectomy/17 + w	I	N/A/≧37w	N/A	N/A	Term infant	DFS 30 months
14		24	8 + w	N/A	N/A	Right salpingo-oophorectomy/13 + w	I	N/A/≧37w	N/A	N/A	Term infant	DFS 18 months
15	Karimi Z M et al. 2008 [17]	26	28w	24 × 16 cm	BEP/29w/2 BEP/Postpartum/2	Peritoneal cytology + right oophorectomy + partial omentectomy/28w Complete omentectomy + ipsilateral lymph node sampling/39w	IIIC	CS/39w		CA125 210 IU/ml; AFP 480 IU/ ml(28 w)	Female, 3100 g Apgar score 9–10 (1st,5th min) Followed up 1.5 years, normal physical and neurological development	Without any evi- dence of tumor recurrence for 1.5 years
16	Daponte A et al. 2008 [18]	33	5w	7 × 7 cm	N/A	Right salpingo-oophorectomy and surgical staging (peritoneal washings, peritoneal and omentum biopsies)/N/A Inspect the peritoneal cavity and biopsies/34w	IA	CS/34w	N/A	AFP 15.94 IU/ml; CA-125 89.6 U/ml(12w)	Healthy infant	After two years no recurrence
17	Poujade O et al. 2008 [19]	36	21w	17.5 cm in diameter	Etoposide and cisplatin/23w/3 Etoposide and cisplatin/Postpartum/2	Left ovariectomy/22w Left salpingectomy/39w	N/A	CS/39w	18 cm in diameter	N/A	3130 g. The Apgar score 10–10-10 (1st,5th,10th min) With normal aspect	In remission six months later
18	Ghaemmaghami F et al. 2009 [20]	25	13w	76 × 45 mm	BEP/N/A/3	Right oophorectomy + biopsy of the left ovary + omentum /21w Partial omen- tectomy + right salpingectomy + perito-neal biopsy + bilateral lymph node sampling/36 w	N/A	CS/36w	N/A	N/A	Male,2000 g Apgar score of 9–10 at 15 min(with normal appearance with a mild hypospadias) Normal physical and neurological development after 8 months of birth	Show no evidence of tumor recurrence after one year
19	Clinkard D J et al. 2011 [21]	23	16w	13 cm	Cisplatinum + etoposide/After abortion/3	Acute surgical exploration/N/A Bilateral oophorectomy + infra-colic omentectomy/N/A	IIIC	Abortion/N/A	N/A	N/A	The fetus spontaneously aborted shortly after the surgery	Six years later alive and delivered a baby
20	Moradan S et al. 2014 [22]	21	18w	18 × 20 cm	N/A	Salpingo-oophorectomy and surgical staging/19w Inspect peritoneal cavity and biopsies were taken from the peritoneum, pelvic wall, left ovary and omentum/38w	IA	CS/38w	N/A	AFP 117 ng/ml; β-HCG/CA125 normal(18w)	Male,2900 g	N/A
21	Luh L et al. 2019 [3]	31	8 + 1w	15 × 15 × 15 cm	BEP/27 + 2w/4	Left oophorectomy + omentectomy + ascites fluid cytology/N/A Total abdominal hysterectomy + salpingectomy sinistra + SOD + lymphadenectomy pelvic bilateral and paraaortic + omentectomy + perit-oneal biopsy/Postpartum	N/A	N/A/40 + 2w	40 × 40 × 40 cm	AFP 699.9 IU/mL; LDH 579 U/L (19 + 5 w)	Female,2700 g Apgar score 7–8 (1st,5th min) Not seen a congenital abnormality	N/A
22	Cochrane E et al. 2020 [23]	26	23w	10.9 × 8.2 × 9.9 cm	BEP/N/A/2 Three cycles of chemotherapy in postpartum(the regimen is not recorded)	Left salpingo-oophorectomy + infracolic omentectomy + left pelvic side-wall biopsy/26w Excision of an anterior peritoneal mass/Postpartum	IIIA	VD/37w	N/A	AFP 1567 ng/mL; CA-125 233.4 U/mL; CA-199 93.1 U/ml(26w)	Female,1790 g Apgar scores 8–9 (1st,5th min) Follow-up 25 months the growth and development normal	Follow-up without further benign or malignant disease
23	Present Case	28	29w	9.7 × 8.5 × 6.4 cm	BEP/Postpartum/4	Left salpingo-oophorectomy + pelvic mass resection + right ovarian biopsy + bilateral pelvic lymph node dissection + para-aortic lymph node sampling + omentectomy/33w	IC	CS/32w	35 × 20 × 15 cm	CA-125 43.4 U/mL; AFP 173.4 ng/mL(30w)	Female,1610 g Apgar scores 9–10-10 (1st,5th,10th)	No evidence of recurrence with 18 months of follow-up after surgery

*AFP* alpha fetoprotein, BEP bleomycin (BLM) + etoposide (VP16) + cisplatin (DDP), *CS* cesarean section, *CT* chemotherapy, *DFS* disease free survival, *GA* gestational age, *β-HCG* β- human chorionic gonadotropin, *N/A* not available*, LDH* lactate dehydrogenase, *VD* vaginal delivery

Tumor marker ^a^refers to the first recorded level of serum tumor markers

Like non-pregnancy patients with immature teratoma, the treatment of pregnant patients with immature teratoma is the same, but more complicated. Operative intervention will increase risk of miscarriage in the first trimester of pregnancy, so surgery should be performed in the second or third trimester when it is possible. Because the survival rate of the fetus is high, immature teratoma in the third trimester of pregnancy is relatively easy to manage. In our study, the pregnant patient was found to have immature teratoma in the third trimester of pregnancy, and she underwent surgery after dexamethasone was given to accelerate fetal lung maturation. Surgery for patients with early-stage immature teratoma is cystectomy or adnexectomy, omentectomy, and peritoneal fluid cytology. Termination of pregnancy followed by removal of tumor mass and chemotherapy is recommended for pregnant patients with advanced-stage immature teratoma (II-IV) before 24 weeks gestational age [3]. A biopsy of tumor mass followed by chemotherapy may be considered for pregnant patients with advanced-stage immature teratoma diagnosed at more than 24 weeks gestational age [24]. Among the 22 cases of immature teratoma in pregnancy reported in the literature, the gestational age data of 17 patients at the time of initial diagnosis are available (Table 1). Of the 17 patients, 11 patients was first diagnosed in the second or third trimesters of pregnancy, and 6 patients in the first trimester. All the patients reported in literature received surgical treatment in the second or third trimesters of pregnancy, and most of these patients underwent unilateral adnexectomy (Table 1). In this study, the patient underwent unilateral adnexectomy, right ovarian biopsy, bilateral pelvic lymph node dissection, para-aortic lymph node sampling, and omentectomy. The patient shows no signs of recurrence after surgery till now.

In addition to surgery, chemotherapy is another important treatment for immature teratomas in pregnancy. Because exposure to teratogens in the first trimester of pregnancy has a higher rate of fetal mortality and congenital malformations, the second and third trimesters of pregnancy are the best time for chemotherapy in pregnant patients. However, chemotherapy in the second and third trimesters of pregnancy may increase the risk of premature delivery, intrauterine growth restriction, stillbirth and low birth weight [25]. It was reported that the chemotherapy drug had no significant side effects on the fetus when its dose was the same as in non-pregnant patient [3, 26]. BEP is the first choice for chemotherapy of immature teratomas, and the recurrence-free survival rate for BEP was reported to be 84% [5]. All of the immature ovarian teratomas in pregnancy reported in the literature received BEP chemotherapy, and no significant post-chemotherapy complications were reported in these fetuses (Table 1). In our study, the pregnant patient received combination chemotherapy with four cycles of BEP regimen, and she shows no sign of recurrence after surgery till now, indicating that immature ovarian teratomas in pregnancy may be sensitive to combination chemotherapy of BEP regimen. Further studies are needed to investigate the possible effects of chemotherapy on outcomes of future pregnancy, long-term neonatal sequelae, and maternal disease progression.

## Conclusions

The incidence of ovarian immature teratomas in pregnancy is very rare. This case report suggests that the benefits and risks of timely treatment for patients and fetuses should be fully assessed by a multidisciplinary team. The early diagnosis, the timing of surgery and chemotherapy, the choice of chemotherapy for BEP will determine the prognosis. Surgery and combination chemotherapy with BEP regimen play a important role in the treatment of immature teratomas in pregnancy, and could gain successful and satisfactory outcomes for mother and fetus.

## Data Availability

The datasets used and/or analysed during the current study available from the corresponding author on reasonable request.

## Abbreviations

MOGCT: Malignant ovarian germ cell tumor
WGA: Weeks gestational age
BEP: Bleomycin-etoposide-cisplatin
MRI: Magnetic resonance imaging
